# Relationship between the LHPP Gene Polymorphism and Resting-State Brain Activity in Major Depressive Disorder

**DOI:** 10.1155/2016/9162590

**Published:** 2016-10-23

**Authors:** Lingling Cui, Xiaohong Gong, Yanqing Tang, Lingtao Kong, Miao Chang, Haiyang Geng, Ke Xu, Fei Wang

**Affiliations:** ^1^Department of Radiology, The First Affiliated Hospital of China Medical University, Shenyang, Liaoning, China; ^2^State Key Laboratory of Genetic Engineering and MOE Key Laboratory of Contemporary Anthropology, School of Life Sciences, Fudan University, Shanghai, China; ^3^Department of Psychiatry, The First Affiliated Hospital of China Medical University, Shenyang, Liaoning, China; ^4^Department of Psychiatry, Yale University School of Medicine, New Haven, CT, USA; ^5^The Research Institute for Brain Functional Imaging, The First Affiliated Hospital of China Medical University, Shenyang, Liaoning, China

## Abstract

A single-nucleotide polymorphism at the LHPP gene (rs35936514) has been reported in genome-wide association studies to be associated with major depressive disorder (MDD). However, the neural system effects of rs35936514 that mediate the association are unknown. The present work explores whether the LHPP rs35936514 polymorphism moderates brain regional activity in MDD. A total of 160 subjects were studied: a CC group homozygous for the C allele (23 individuals with MDD and 57 controls) and a T-carrier group carrying the high risk T allele (CT/TT genotypes; 22 MDD and 58 controls). All participants underwent resting-state functional magnetic resonance imaging (rs-fMRI) scanning. Brain activity was assessed using the amplitudes of low-frequency fluctuations (ALFF). MDD patients showed a significant increased ALFF in the left middle temporal gyrus and occipital cortex. The T-carrier group showed increased ALFF in the left superior temporal gyrus. Significant diagnosis × genotype interaction was noted in the bilateral lingual gyri, bilateral dorsal lateral prefrontal cortex (dlPFC), and left medial prefrontal cortex (mPFC) (*P* < 0.05, corrected). Results demonstrated that MDD patients with LHPP rs35936514 CT/TT genotype may influence the regional brain activity. These findings implicate the effects of the rs35936514 variation on the neural system in MDD.

## 1. Introduction

The CONVERGE consortium has obtained a large number of samples (including 5,303 cases of MDD and 5,337 controls, all Chinese subjects) for inclusion in genome-wide association studies (GWAS). SNP rs35936514 in the LHPP gene has been reported to be significantly associated with MDD at a genome-wide level [1]. LHPP encodes an enzyme known as phospholysine phosphohistidine inorganic pyrophosphate phosphatase (LHPP), which was originally purified from swine brain tissue [2] and shows high levels of expression in the brain [3, 4]. This region of the genome has been implicated in the etiology of MDD using a combination of linkage and association analysis [4]. The associations have been shown to be dependent on HTR1A genotype. LHPP or a product of a collinear brain-specific transcript, therefore, may interact with HTR1A in the pathogenesis of major depression.

A recent study has extended the findings of previous literature by supporting the role of LHPP in risk for MDD [5]. A single study has shown the expression of LHPPase to be associated with thyroid function [6], which could be interesting given that thyroid disorder is thought to mediate the functional regulation of MDD patients [7]. A link between thyroid function and depression was reported in the late 1960s [8]. Any impairment of thyroid function supply to the developing CNS causes severe changes to the overall architecture and function of human brain, leading to various neurological dysfunctions [9–12]. A large number of clinical trials later confirmed this finding [12–15].

Recently, genetic imaging has been used to investigate the association of genetic variation with neuroimaging endophenotypes [16]. Genetic imaging is an emerging field that is rapidly identifying genes that influence the brain, cognition, or risk for diseases. Along with clinical diagnosis, brain imaging offers fine-grained measures that can be subjected to genetic analysis. It has fostered new enthusiasm in depression research, because this approach allows for assessing the neural impact of candidate genes in vivo and thus provides for a new level of evidence [17, 18]. Resting-state functional magnetic resonance imaging (rs-fMRI) is often used to examine mental disorders [19]. The amplitude of low-frequency fluctuations (ALFF) is thought to detect the intensity of spontaneous neural activity at rest. It has been considered a reliable and sensitive measure to study both healthy and clinical populations [20–22].

The effects of the neural system and their association with LHPP variation in the context of MDD are not yet understood. Identifying previously unknown physiological pathways can open new avenues for the development of novel depression drugs. In the present study, the association of LHPP rs35936514 with the resting-state brain activity was measured using ALFF. It is here hypothesized that variations in rs35936514 may influence spontaneous brain activity at rest in MDD patients.

## 2. Methods

### 2.1. Participants

A total of 160 Chinese subjects were studied. 45 MDD patients (mean age 27.13 ± SD  12.65 years, range 13–59 years, 71% female) were recruited from the outpatients at the Department of Psychiatry, the First Hospital of China Medical University, and the Mental Health Center of Shenyang. The diagnosis of MDD was confirmed by 2 trained psychiatrists using the Structured Clinical Interview for DSM-IV Disorders (SCID) [23]. Scores on the 17-item Hamilton Rating Scale for Depression (HAMD) were obtained from each participant except for one, who did not complete these evaluations [24]. Here, 115 healthy control (HC) subjects (mean age 33.96 ± SD  14.25 years, range 7–58 years, 57% female) without any personal history of any Axis I disorder or first-degree family member with a major mood or psychotic disorders were recruited through surrounding communities. Exclusion criteria for both groups included history of neurological disease, loss of consciousness ≥5 min, any medical condition that might affect neurovascular function including hypertension, current substance abuse, or dependence, and having contraindications for MRI. All participants provided written informed consent after receiving a complete description of the study as approved by the Institutional Review Board of the China Medical University.

### 2.2. Genotyping

Ten-milliliter blood samples were collected from participants for DNA extraction. The genotypes of the SNP at rs35936514 were determined using Sanger sequencing method. Subjects were further divided into two groups: a CC group homozygous for the C allele (23 MDD, 57 HC; mean age = 34.06 ± 14.45 years, 60% female) and risk T-carrier group (CT/TT genotypes; = 22 MDD, 58 HC; mean age = 30.02 ± 13.57 years, 61% female). Minor allele frequencies were 18%. Genotype frequencies were consistent with Hardy-Weinberg equilibrium expectation (MDD: *χ* ^2^ = 2.894, *P* = 0.08; HC: *χ* ^2^ = 2.52, *P* = 0.11).

### 2.3. MRI Acquisition

The MRI data were acquired using a GE Signa HDX 3.0T MRI scanner (General Electric, US) in the Department of Radiology of the First Hospital of China Medical University. Soft pads and earplugs were used when scanning to restrict head motion and reduce scanner noise. Participants were asked to relax with their eyes closed but remain awake throughout the resting-state scan. Three-dimensional T1-weighted images were obtained using a Fast Spoiled Gradient-Echo (FSPGR) sequence: repetition time (TR) = 7.1 ms, echo time (TE) = 3.2 ms, field of view (FOV) = 24 cm × 24 cm, flip angle = 15°, matrix = 240 × 240, slice thickness = 1 mm, and no gap. Functional images were acquired using a gradient echo planar imaging (EPI): TR = 2000 ms, TE = 30 ms, FOV = 24 cm × 24 cm, flip angle = 90°, matrix = 64 × 64, slice thickness = 3 mm, no gap, and slices = 35.

### 2.4. MRI Data Processing

Resting-state fMRI data were preprocessed using SPM8 (http://www.fil.ion.ucl.ac.uk/spm/). The first 10 volumes of each subject were discarded to allow participants to adapt to the scanning environment. The remaining data were preprocessed including slice timing, head motion correction, and spatial normalization to the standard Montreal Neurological Institute (MNI) space (resampling to 3 × 3 × 3 mm^3^) [25]. Subsequently, the images were spatially smoothed with a 6 mm full-width at half-maximum (FWHM) Gaussian kernel. Participants with head motion less than 3.0 mm in any dimension or 3° rotation at any point during the course of the scan were included for further analysis.

Subsequent data preprocessing included removal of linear trends and temporal filtering (band pass, 0.01–0.08 Hz) to reduce the effects of low-frequency drift and high-frequency noise [26]. The calculations were performed in the same manner as in previous studies [27]. The square root was calculated at each frequency of the power spectrum and averaged square root was between 0.01 and 0.08 Hz. This averaged square root was termed as ALFF [22]. For standardization, the ALFF of each voxel was divided by the global mean ALFF value within a brain mask [19].

Resting-state fMRI Data Analysis Toolkit (REST) (http://www.restfmri.net/) was performed for further data processing and ALFF analysis.

### 2.5. Statistical Analysis

Demographic data (sex, age, education, and HAMD) were analyzed using Chi-square tests and two-way analysis of variance (ANOVA) with diagnostic group (HC, MDD) and genotype group (CC, CT/TT) as between subject factors. A two-sample *t*-test was used to compare the durations of illness across genotypes within the MDD group. All statistical analyses were performed using SPSS 13.0 software (SPSS Inc.). All statistical thresholds were set at *P* value < 0.05.

A voxelwise ANOVA (2 × 2 ANOVA: diagnosis × genotypes) was used to determine the effects of diagnosis and genotype on ALFF values, and age and sex were considered as covariates. Post hoc *t*-test was used to explore the details of the main effects and interactions. Significant interactions were interpreted using graphical displays. Correction for multiple comparisons was based on Monte Carlo simulation [AlphaSim, Analysis of Functional Neuroimages (AFNI) [28], cluster size > 3321 mm^3^]. Pearson correlation analyses and Spearman correlation analyses were performed to determine the correlation of ALFF values between regions showing significant differences with the HAMD scores, education, and illness duration in the two diagnostic groups.

## 3. Results

### 3.1. Participant Characteristics

There was no significant effect of diagnosis, genotype, or interaction between diagnosis and genotype for age, sex, or education. The effect of diagnosis in HAMD was significant, with significantly higher HAMD scores in the MDD group than in the HC group. Genotype showed no significant effects on HAMD. Two-sample *t*-tests showed no difference in the duration of illness between the MDD subgroups (Table 1).

### 3.2. ALFF Analyses

The influence of diagnosis and genotypes on ALFF in the CC and CT/TT genetic subgroups in the MDD and control subjects is listed in Table 2.

**Figure 1:**
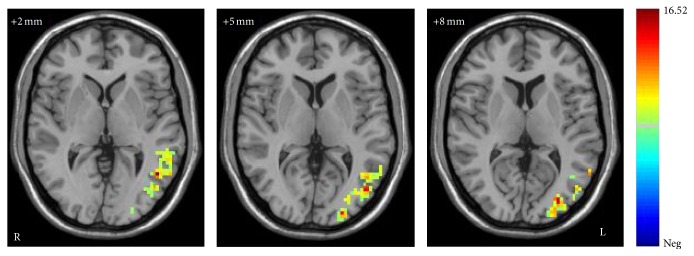
The main effect of diagnostic groups on ALFF in the MDD and HC. The axial images (MNI coordinate *z* = +2 mm, +5 mm, and +8 mm) display the regions in the left middle temporal gyrus and left occipital cortex that show increased ALFF in patients with major depressive disorder (MDD), compared to healthy controls (HC) at rest (*P* < 0.05 by AlphaSim correction, and 123 voxels minimum). Color bar represents the range of *F* values.

**Figure 2:**
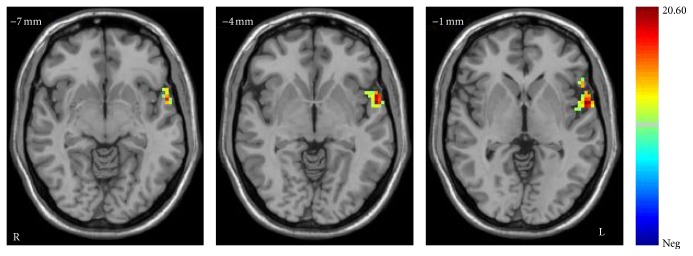
Main effect of genotypes on ALFF in the MDD and HC. The axial images (MNI coordinate *z* = −7 mm, −4 mm, and −1 mm) display the regions in the left superior temporal gyrus that show increased ALFF in the T-carrier group (*P* < 0.05 by AlphaSim correction, and 123 voxels minimum). Color bar represents the range of *F* values.

After performing a two-way ANOVA on the ALFF maps, a significant main diagnosis group effect was observed (*F* = 56.39, *P* < 0.001, corrected). MDD patients showed significantly increased ALFF in the left middle temporal gyrus and left occipital cortex compared with individuals in HC group (Figure 1). There was also a significant main effect of genotype (*F* = 31.80, *P* < 0.001, corrected). The T-carrier group showed increased ALFF in the left superior temporal gyrus (Figure 2).

**Figure 3:**
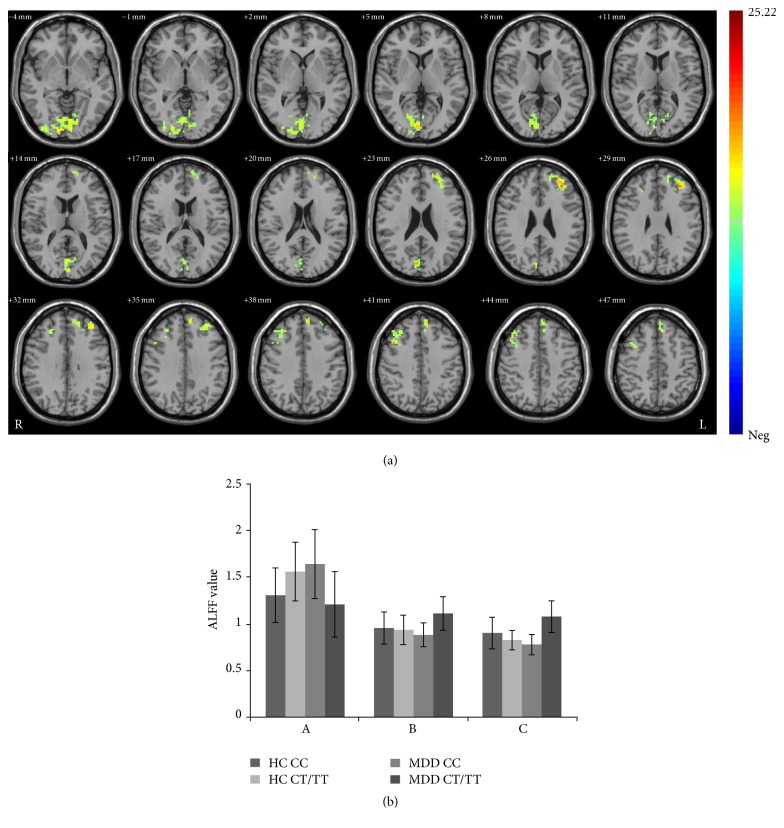
Interaction between rs35936514 genotypes and diagnosis. (a) Clusters of significance for the interaction effect in the bilateral lingual gyri, bilateral dlPFC, and left mPFC (*P* < 0.05 by AlphaSim correction, and 123 voxels minimum). Color bar represents the range of *F* values. (b) Interaction graph showing the ALFF value was (A) decreased in bilateral lingual gyri and (B) increased in the left dlPFC and mPFC and (C) right dlPFC, in MDD patients with CT/TT genotype.

Significant diagnosis by genotype interaction was noted (*F* = 22.24, *P* < 0.001, corrected). Among patients with MDD, the T-carrier group had significantly lower ALFF values in the bilateral lingual gyri than the CC group (*F* = 2.92, *P* = 0.006). Within-genotype comparisons showed that T-carriers with MDD had significantly lower ALFF in the bilateral lingual gyri than healthy controls who were T-carriers (*F* = 4.82, *P* = 0.02). In addition, ALFF were highest in the brain regions of the bilateral dorsal lateral frontal cortex (dlPFC) and left medial prefrontal gyrus (mPFC) in MDD rs35936514 T-carriers compared to MDD rs35936514 CC homozygotes and both control genotype groups (*P* < 0.001) (Figure 3).

Correlation analyses did not show any significant associations between ALFF and HAMD scores, illness duration, or education in MDD or HC groups.

## 4. Discussion

Based on the large number of samples studied on LHPP rs35936514 associated with MDD in Chinese subjects [1], it was here noted that LHPP was associated with thyroid disorders, which may affect depression [6]. Multiple studies have reported that both hypo- and hyperthyroidism may potentially increase the risk of cognitive impairment and neurodegeneration. Hyperthyroidism might cause depression as a result of thyrotoxicosis [29]. The association between hypothyroidism and depression might be explained by higher rates of hypercortisolism in depression [30], which might lead to changes in the hypothalamic-pituitary-thyroid/adrenal (HPT/HPA) axes [31]. Dysfunctional HPT/HPA axes regulation might be a trait in MDD patients, producing changes in the monoaminergic pathways that modulate hormonal responses [32]. Autoimmune thyroiditis which is marked by the presence of thyroid antibodies with normal or abnormal thyroid hormone level is also a major cause for MDD. A bidirectional association between depression and the immune system has been also reported [33, 34]. Depression has been linked with increased inflammatory markers and depression risk alleles have been found to be associated with regulating genes of the immune response [35]. However, the current work did not show apparent relationship between LHPP, thyroid disorders, and MDD. Our present study may provide a new imaging genetics approach to explore their relationships.

In order to assess the effects on the neural system associated with LHPP variation and MDD, the current work demonstrated that LHPP rs35936514 SNP polymorphism may influence the spontaneous brain activity in individual subjects with MDD and also healthy controls.

The spontaneous low-frequency fluctuations (LFF) of blood oxygenation level-dependent (BOLD) signals at rest have been identified as a biological measure of baseline spontaneous activity in the brain [21, 22, 36]. Abnormal ALFF can reflect pathophysiological states in the regional brain area and may help to locate specific impaired brain regions during the resting state [21]. As an additional advantage, ALFF can be used to assess neuronal activity within the entire brain [21]. In particular, by rs-fMRI, the cortico-limbic-striatal circuits (including the PFC, hippocampus, amygdala, and striatum) have been implicated in the dysfunctional regulation in MDD [37].

The current study showed significant interactions between diagnosis and genotype in the bilateral lingual gyri, bilateral dlPFC, and left mPFC. The alterations of ALFF in the MDD T-carrier group suggest that the risk T allele is associated with abnormal spontaneous activity in MDD. More research is needed to identify the mechanisms by which these regional activities in individuals with MDD may be more vulnerable to the effects of variations in rs35936514, which render them more vulnerable to genetic liability. However, it remains unknown when and how this interaction could change ALFF, although many factors could influence the status of MDD [38–40], which are well established as a risk for MDD [41]. Furthermore, expression of multiple genes involved in MDD [42], and their interaction with the LHPP genotype could make a change in the neural circuits of MDD patients. The parts of the brain in the mPFC are believed to be the neural site of self-referential processing, like the anterior node of the default mode network (DMN). Alterations to the mPFC functional networks are involved in the development of MDD [43, 44]. Altered resting state in the dlPFC of MDD could adversely affect activity during emotional or cognitive regulation. The lingual gyrus has been reported to participate in the visual recognition network and to play a role in the perception of emotions [45, 46]. Many studies have reported decreased activity in the lingual gyrus [45, 47, 48] in patients with MDD. Different brain regions were responsible for changes in ALFF in patients with MDD. This may be attributable to different roles and levels of expression of the LHPP gene in different regions of the brain, different structural or functional bases (activities) of different brain regions, the combined effects of the T-carrier genotype, and other unknown factors [38].

In this study, independent of genotype, increased ALFF was observed in the left middle temporal gyrus and left occipital cortex among MDD participants compared to that among HCs. The increased spontaneous activity in the temporal and occipital lobe within MDD patients was consistent with the findings of rs-fMRI studies examining depression [49–51].

Genotype was found to have an effect on ALFF in the left superior temporal gyrus. It was higher among T-carriers of the LHPP rs35936514 polymorphism in both the HC and MDD groups than in CC individuals within the respective diagnostic groups. It is here suggested that the MDD rs35936514 T-carriers may function as one of several factors that can be used to diagnose MDD. It is possible that variation in LHPP may contribute to the different ALFF values in temporal lobe in individuals with or at risk for MDD. In this way, the subgroup with MDD that carries the T allele may be a risk factor to abnormal brain activity. However, exploratory analyses did not reveal significant correlations between ALFF values in regions of significant group differences and clinical factors within each group. We speculate that the relative small sample size or other clinical factors may limit our ability to detect the relation.

Other LHPP SNPs have also been associated with MDD. One LHPP SNP, rs11245316, was found to confer risk of MDD by QTL-specific association analysis [5]. However, these data do not provide preliminary indications of how genetic variation in high-risk regions influences susceptibility to neural circuits abnormalities in patients with MDD.

The present work has some limitations. First, many SNPs and genes implicated in MDD were not included in the study. Gene-gene interaction studies and even pathway studies are needed to search for valid MDD-associated genetic and neuroimaging biomarkers. Second, owing to the small sample size, the study placed individuals with CT and TT genotype in a single group. As a result, no analysis of the ALFF values among 3 genotype groups could be performed, and the effect of the risk T allele could not be established as dominant or codominant. A larger-sample study should be performed to further investigate the effect of genes.

## 5. Conclusions

In conclusion, the present study demonstrated for the first time that LHPP rs35936514 CT/TT genotype may affect regional brain activity in MDD patients. This suggests that the influence of LHPP variation may be one mechanism that contributes to the neural circuits of MDD.

## Figures and Tables

**Table 1:** Demographics and clinical data of participants.

	MDD (*n* = 45)		HC (*n* = 115)			*P*
	CC (*n* = 23)	CT/TT (*n* = 22)	CC (*n* = 57)	CT/TT (*n* = 58)		
Gender (male/female)	7/16	6/16	25/32	25/33	*χ* ^2^ = 3.43	0.18
Age (years), mean ± SD	29.91 ± 12.97	31.22 ± 11.91	32.73 ± 14.78	32.22 ± 13.61	*F* = 1.21	0.65
Education (years), mean ± SD	11.27 ± 2.41	11.19 ± 2.89	13.35 ± 4.08	12.55 ± 4.47	*F* = 0.40	0.52
Duration of illness (months), mean ± SD	11.33 ± 16.78	8.40 ± 9.84	NA	NA		0.22
HAMD score, mean ± SD	20.74 ± 7.73	21.81 ± 6.95	1.18 ± 2.20	1.12 ± 2.24	*F* = 11.30	0.000

MDD, major depressive disorder; HC, healthy controls; SD, Standard Deviation; HAMD, Hamilton Depression Rating Scale.

**Table 2:** Clusters exhibiting the influence of groups and genotypes on ALFF in the CC and CT/TT genetic subgroups in the MDD and HC.

Brain area	BA	Cluster size	Peak MNI coordinates			Peak *F* value
			*X*	*Y*	*Z*	
*Main effect of diagnostic groups*						
Left middle temporal gyrus/left occipital cortex	19	194	−48	−60	3	56.39
*Main effect of genotypes*						
Left superior temporal gyrus	22	124	−57	3	0	31.80
*Diagnostic groups × genotypes interaction*						
Bilateral lingual gyri	17/18	422	9	−96	−3	22.24
Left dorsal lateral prefrontal cortex/left medial prefrontal cortex	10	167	−33	39	27	33.96
Right dorsal lateral prefrontal cortex	6/9	152	39	24	54	36.67

These findings correspond to a corrected *P* < 0.05 by AlphaSim correction. BA: Brodmann's area. Cluster size is in mm^3^.
